# Elucidating population-wide mycobacterial replication dynamics at the single-cell level

**DOI:** 10.1099/mic.0.000288

**Published:** 2016-06

**Authors:** Jacoba M. Mouton, Sophie Helaine, David W. Holden, Samantha L. Sampson

**Affiliations:** ^1^​ DST/NRF Centre of Excellence for Biomedical Tuberculosis Research/SA MRC Centre for Tuberculosis Research, Division of Molecular Biology and Human Genetics, Faculty of Medicine and Health Sciences, Stellenbosch University, Cape Town, South Africa; ^2^​ MRC Centre for Molecular Bacteriology and Infection, Imperial College London, London, UK

**Keywords:** Mycobacterium tuberculosis, replication, persister, heterogeneity, macrophage

## Abstract

*Mycobacterium tuberculosis* infections result in a spectrum of clinical outcomes, and frequently the infection persists in a latent, clinically asymptomatic state. The within-host bacterial population is likely to be heterogeneous, and it is thought that persistent mycobacteria arise from a small population of viable, but non-replicating (VBNR) cells. These are likely to be antibiotic tolerant and necessitate prolonged treatment. Little is known about these persistent mycobacteria, since they are very difficult to isolate. To address this, we have successfully developed a replication reporter system for use in *M. tuberculosis*. This approach, termed fluorescence dilution, exploits two fluorescent reporters; a constitutive reporter allows the tracking of bacteria, while an inducible reporter enables the measurement of bacterial replication. The application of fluorescence single-cell analysis to characterize intracellular *M. tuberculosis* identified a distinct subpopulation of non-growing mycobacteria in murine macrophages. The presence of VBNR and actively replicating mycobacteria was observed within the same macrophage after 48 h of infection. Furthermore, our results suggest that macrophage uptake resulted in enrichment of non- or slowly replicating bacteria (as revealed by d-cycloserine treatment); this population is likely to be highly enriched for persisters, based on its drug-tolerant phenotype. These results demonstrate the successful application of the novel dual fluorescence reporter system both *in vitro* and in macrophage infection models to provide a window into mycobacterial population heterogeneity.

## Introduction

Despite recent data indicating a worldwide decline in tuberculosis (TB) mortality and incidence (WHO, 2015), the disease continues to present serious public health challenges. The first key challenge is posed by the large number of individuals with latent *Mycobacterium tuberculosis* infection, an estimated one-third of the world’s population (Hartman-Adams *et al.*, 2014). This represents an ongoing infectious disease threat which is particularly relevant in high-incidence settings where human immunodeficiency virus (HIV) co-infection is also common. In HIV co-infected individuals, there is a substantially increased risk of progression to active disease (WHO, 2015). A second significant obstacle lies in the requirement for very long and complex treatment regimens, which are required to achieve complete sterilization of *M. tuberculosis *infections. This is thought to be driven by so-called ‘persister' or ‘dormant' populations of mycobacteria, which exhibit a drug-tolerant phenotype (Zhang *et al.*, 2012). While there are varying definitions of the term ‘persister', here we use it to refer to non- or slowly replicating, drug-tolerant bacteria.

The existence of drug-tolerant populations of persistent bacteria was first postulated in the 1940s, following the observation that *Staphylococcus aureus* surviving antibiotic treatment were phenotypically and reversibly tolerant, rather than genetically resistant (Bigger, 1944). Since then, the phenomenon has been well described in *Escherichia coli*, and numerous other bacteria have been shown to form persisters (Cohen *et al.*, 2013; Helaine & Kugelberg, 2014). Several mediators of persistence have been identified, with toxin–antitoxin modules emerging as key players in formation and maintenance of persister populations (Cohen *et al.*, 2013; Helaine & Kugelberg, 2014; Maisonneuve *et al.*, 2011). Although limited studies have been carried out in *M. tuberculosis*, these are mostly consistent with findings in other bacteria (Dhar & McKinney, 2010; Keren *et al.*, 2011; Manina *et al.*, 2015).

A commonly accepted notion is that bacterial persister populations are non-replicating, which contributes to their drug-tolerant phenotype (Helaine & Kugelberg, 2014), although this assumption has been challenged by recent studies (Adams *et al.*, 2011; Manina *et al.*, 2015; Wakamoto *et al.*, 2013). Understanding the physiological state of persister mycobacteria and their role in clinically latent *M. tuberculosis *infection has important implications for TB treatment and prevention. However, our understanding of this physiological state is hampered by a paucity of suitable tools to identify, isolate and characterize non- or slowly replicating mycobacteria.

The nature of bacterial persisters renders them difficult to isolate and characterize. In the first instance, they are likely to undergo very limited (if any) replication. This makes them difficult to recover in their viable, but non-replicating or non-growing, metabolically active state, while still reflecting relevant physiology. Secondly, based on data from other bacteria and indirect studies in mycobacteria, they are likely to only be present in very low numbers. We therefore require culture-independent methods which will allow us to enrich for, and isolate, persistent bacteria without perturbing their physiological state. Given the emerging appreciation of the heterogeneous nature of bacterial populations, it is also important to be able to study single cells at the population-wide level. Recent studies have exploited the power of high-resolution technologies such as microfluidics and flow cytometry to enable the rapid measurement of the physiological state of single bacteria within large populations (Aldridge *et al.*, 2012; Balaban *et al.*, 2004; Maglica *et al.*, 2015; Manina *et al.*, 2015; Roostalu *et al.*, 2008; Wakamoto *et al.*, 2013). Helaine *et al.* (2010, 2014)successfully applied the latter to measure fluorescence dilution (FD) in *Salmonella *Typhimurium, using a dual fluorescence replication reporter system. These studies revealed that macrophage uptake induces a non-replicating population of *Salmonella*, leading to important insights into the characteristics of non-replicating persistent bacteria *in vivo*. As we predict that similar mechanisms are important during *M. tuberculosis *infection, in this study we aimed to develop and validate a similar replication reporter system for *M. tuberculosis. *This has been applied to provide insights into population-wide mycobacterial replication dynamics at the single-cell level.

## Methods

### Bacterial strains and culture.

All reagents were purchased from Sigma-Aldrich, unless otherwise specified. Bacterial strains utilized in this study are listed in Table 1. Electrocompetent *E. coli *DH10B used for plasmid selection and propagation was obtained from Invitrogen. *Mycobacterium smegmatis* mc^2^155 was obtained from the American Type Culture Collection (ATCC 700084). The origin and construction of *M. tuberculosis *Δ*leuD*Δ*panCD* was as previously reported (Sampson *et al.*, 2004).

**Table 1.** Plasmids and strains

Plasmid/strain	Description	Source
pST5552	*hsp60(ribo)-egfp* (inducible EGFP under control of theophylline-inducible riboswitch), Kan^R^, episomal	Seeliger *et al.* (2012), Addgene plasmid number 36255
pCHARGE3	Psmyc-TurboFP635, Hyg^R^, episomal	Carroll *et al.* (2010), Addgene plasmid number 24658
pSTCHARGE3	*hsp60(ribo)-turboFP635 *(inducible TurboFP635 under control of theophylline-inducible riboswitch), Kan^R^, episomal	This study
pTiGc	*hsp60(ribo)-turboFP635 hsp60*-*gfp*, Kan^R^, episomal	This study
*E. coli *DH10B	Cloning host	Invitrogen
*M. smegmatis* mc^2^155	Non-pathogenic, fast-growing model organism	ATCC 700084
*M. tuberculosis *Δ*leuD* Δ*panCD*	Double leucine and pantothenate auxotroph	Sampson *et al.* (2004)

*E. coli* was cultured in lysogeny broth (LB) with appropriate antibiotic supplementation according to standard protocols at 37 °C, with shaking, or on LB agar at 37 °C. Liquid cultures of mycobacterial strains were grown in 7H9 supplemented with 10 % oleic acid–albumin–dextrose–catalase (OADC; Becton Dickinson), 0.2 % (v/v) glycerol and 0.05 % (v/v) Tween 80 (7H9-OGT), with appropriate antibiotic supplementation, at 37 °C, with shaking. Electro-competent mycobacteria were prepared and transformed as described by Snapper *et al.* (1990). Solid media cultures of mycobacteria were grown on 7H10 agar supplemented with 10 % OADC, 1 % (v/v) glycerol and appropriate antibiotics at 37 °C. Additionally, for c.f.u. enumeration only, *M. smegmatis* was cultured on LB agar. To induce the expression of proteins under control of the ribo switch-based promoter, theophylline was added at 2 mM.

For testing the stability of fluorescent reporter proteins, cultures were washed, then passaged twice through PBS with 0.05 % tyloxapol and 2 mM theophylline for 24 h each time. Cultures were then transferred to PBS with 0.05 % tyloxapol (without theophylline) in the presence of 30 µg ml^−1^ of the protein synthesis inhibitor chloramphenicol.

### Plasmid constructs.

Plasmids used and constructed in this study are listed in Table 1. pST5552 (carrying EGFP under control of the theophylline-inducible riboswitch promoter) (Seeliger *et al.*, 2012) and pCHARGE3 (encoding the far-red fluorescent protein, TurboFP635, under control of the P_s*myc*_promoter) (Carroll *et al.*, 2010) were obtained from Addgene (Table 1). The pSTCHARGE3 plasmid (carrying inducible TurboFP635) and pTiGc plasmid (carrying inducible TurboFP635 and constitutive GFP) were generated in this study. Briefly, the TurboFP635 ORF was PCR amplified from pCHARGE3 with primers incorporating *Eco*RI and *Hin*dIII sites (forward primer: 5′-CGATCCGAATTCAGGAGGGAATTC-3′, reverse primer: 5′-ATCGATAAGCTTTTACGAGTG-3′). Following *Eco*RI/*Hin*dIII restriction digestion, the TurboFP635 PCR fragment was cloned into the corresponding sites in pST5552, replacing EGFP and inserting TurboFP635 under control of the riboswitch promoter, to generate pSTCHARGE3. The pTiGc plasmid was subsequently generated by excising *hsp60*-*gfp* from pMV306hspGFP (Parker & Bermudez, 1997) with *Not*I, and cloning into *Not*I-restricted pSTCHARGE3.

### Mammalian cell culture.

RAW264.7 cells (ATCC TIB-71) were cultured in Dulbeco’s Modified Eagle’s Medium (DMEM), supplemented with 10 % heat-inactivated FCS at 37 °C in 5 % CO_2_. Cells were passaged every 2–4 days. For infections, cells were seeded at 5×10^5^ cells per well in 24-well plates. Where required for microscopy, 14 mm glass coverslips were sterilized and added to appropriate wells prior to seeding. The following day, medium was replaced with fresh DMEM/10 % FCS. To prepare mycobacteria for infection, cultures were filtered through 40 μm cell strainers, then briefly sonicated in an ultrasonic bath (UC-1D; Zeus Automation) at 37 kHz for 12 min to disperse clumps (these conditions have previously been shown to reduce clumping without affecting bacterial viability). Bacteria were then washed with PBS/0.05 % Tween 80 (PBS-T) prior to centrifugation and resuspension in DMEM/10 % FCS. Bacteria were added to macrophages at a 5 : 1 ratio, and incubated at 37 °C in 5 % CO_2_ for 3 h. Following the uptake period, cells were washed once with PBS before the medium was replaced with DMEM/10 % FCS containing 100 U penicillin/streptomycin. The cells were then incubated at 37 °C in 5 % CO_2 _for 1 h to kill any non-phagocytosed, extracellular bacteria. Cells were washed three times before adding fresh DMEM/10 % FCS. To recover mycobacteria for c.f.u. determination and flow cytometry, macrophages were lysed by the addition of sterile distilled water followed by pipetting. C.f.u. determination was performed by serial dilution plating of lysates onto LB agar (for *M. smegmatis*) or 7H10 agar (for *M. tuberculosis*). To induce or maintain the expression of bacterial proteins under control of the riboswitch-based promoter, theophylline was added to mammalian cell cultures at 1 mM. To ensure that dilution of fluorescence only started intracellularly, after macrophage uptake, macrophages were pre-incubated with 1 mM theophylline, and theophylline was maintained in the cultures up to and including the antibiotic incubation step (this was defined as *t*=0).

### Persister assay.

To assess whether internalization of *M. tuberculosis* in macrophages enriches for drug-tolerant bacteria (persisters), murine macrophages were infected with pre-induced *M. tuberculosis *as described above. Following uptake, penicillin/streptomycin treatment and PBS washes were performed as described above. At specified time points, bacteria were either recovered from infected macrophages and treated with d-cycloserine (DCS), or DCS-treated while still within macrophages, to select for drug-tolerant persisters. More specifically, intracellular bacteria recovered from lysed infected macrophages were washed once in PBS and incubated in 7H9 containing 100 µg DCS ml^−1^ (Keren *et al.*, 2011) at 37 °C for 4 days. C.f.u. determination was performed by serial dilution plating of lysates onto 7H10 agar. Untreated controls were included for both c.f.u. and flow cytometry assessments. The fold increase in drug-tolerant bacteria induced by internalization in macrophages was quantified as previously described (Helaine *et al.*, 2014), by calculating the ratio between the percentage surviving macrophage-exposed population and the percentage surviving *in vitro*-cultured bacteria. Here, percentage surviving refers to the bacteria surviving DCS treatment (non-replicating persisters). To assess population heterogeneity and persister enrichment by flow cytometry, macrophages with intracellular bacteria were incubated with DMEM/10 % FCS containing 100 µg DCS ml^−1^, without theophylline, at 37 °C in 5 % CO_2_ for 4 days.

### Flow cytometry sample preparation, acquisition and analysis.

Bacteria from *in vitro *culture media (7H9-OGT or DMEM/10 %FCS) were gently sonicated as described above. Sonicated bacteria from *in vitro *cultures, or bacteria recovered from lysed macrophages, were pelleted, fixed in 4 % formaldehyde for 30 min, then washed twice in PBS-T. Samples not analysed immediately were stored in PBS-T in the dark at 4 °C, then pelleted, resuspended in PBS, and filtered immediately prior to use. Samples were analysed using a LSRFortessa flow cytometer (Becton Dickinson). In addition to forward scatter (FSC) and side scatter (SSC), GFP fluorescence intensity was captured by excitation at 488 nm, using a 530/30 filter, and TurboFP635 fluorescence intensity was captured by excitation at 561 nm, using a 610/20 filter. For each experiment, compensation was performed using unlabelled and single-colour controls. For samples from *in vitro *liquid cultures, 10 000 to 30 000 events were captured, while 30 000 events were captured for bacterial samples recovered from macrophages.

Flow cytometry data were analysed using FlowJo vX.0.07r2 software. A primary gate was set based on FSC/SSC properties, following which the GFP-positive (live) population was gated, then analysed to determine the geometric mean of the TurboFP635 fluorescence intensity (Fig. S1, available in the online Supplementary Material). To calculate the number of generations, based on fluorescence intensity data, the extent of bacterial replication (*F*=fold replication) was first determined by the ratio *Y*_0_/*Y_t_*, where *Y* is the geometric mean of red (TurboFP635) fluorescence intensity at a specific time. The number of generations was in turn calculated from the formula *F*=2*^N^*. The number of generations as determined by OD measurements or c.f.u. data was calculated similarly, except that in this case *F*=*Y_t_*/*Y_o_*. Generation times are expressed as mean±sd.

### Microscopy.

Bacteria from *in vitro *culture medium were sonicated and formaldehyde fixed as described above, then washed with PBS and mounted onto clean microscope slides using Prolong Gold (Invitrogen). Similarly, infected macrophages on coverslips were fixed with 4 % formaldehyde for 30 min, washed with PBS and mounted onto clean microscope slides using Prolong Gold (Invitrogen). Cells were observed using a confocal microscope (LSM 780; Carl Zeiss) equipped with a GaAsp detector, using a plan-apochromat ×63/1.4 oil DIC M27 objective. Samples were excited with a 488 and 561 nm laser, using 490–516 nm and 585–696 nm filters for green and red fluorescence, respectively. Transmitted light images for infected macrophages were collected using a confocal microscope equipped with a transmitted light detector. Images were acquired through *z*-stack acquisition, with an increment of 0.500 µm between image frames, and displayed as maximum-intensity projections. Zen imaging software (Zen SP1 2012, Black edition, version 8.1.0.484) was used to view and process images.

### Statistical analysis.

Statistical analysis was carried out using GraphPad Prism v6.04 software.

## Results

### Construction of replication reporter

**Fig. 1.**
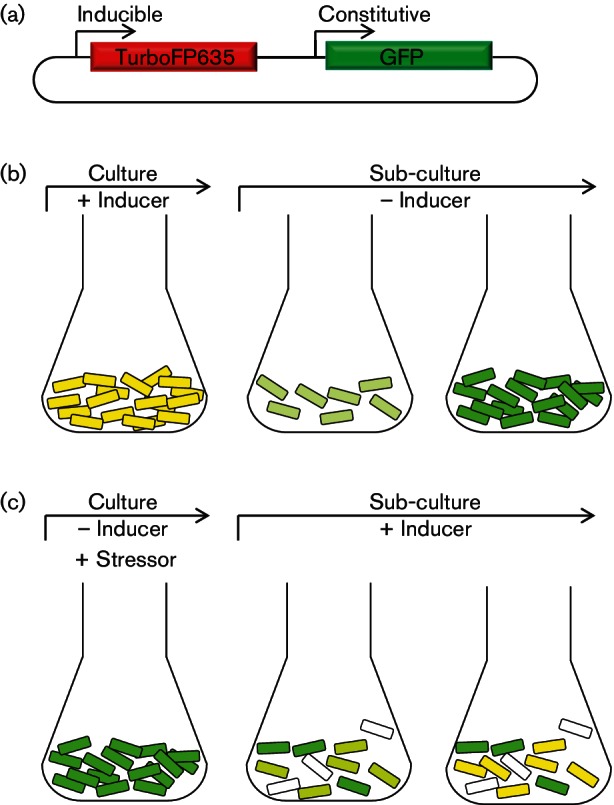
Principle underlying the inducible dual reporter system for probing bacterial replication. (a) Schematic representation of pTiGc reporter plasmid, with GFP under control of the constitutive *hsp60 *promoter, and TurboFP635 under control of the theophylline-inducible, riboswitch-based reporter (Seeliger *et al.*, 2012). (b) Application of FD to monitor replication dynamics, where inducible fluorescent signal is diluted as bacteria replicate. (c) Application of FD to probe metabolic responsiveness following exposure to a stressor (e.g. antibiotic). In this case, viable bacteria would be expected to exhibit green fluorescence, metabolically active bacteria will exhibit both red and green fluorescence following induction, while dead bacteria will not fluoresce.

The ability to reliably measure bacterial replication dynamics using FD requires two sufficiently bright and stable, spectrally distinct, fluorescent reporter proteins, and a tightly regulated promoter. This approach relies on the constitutive expression of one reporter protein as a marker of cells, in combination with an inducible reporter protein (Fig. 1a), preferably with a large dynamic range of dilution, which acts as a marker of replication (Fig. 1b). Alternatively, the inducible reporter can be exploited as a proxy for metabolic activity, specifically translational activity (Fig. 1c). To this end, we made use of GFP (Inouye & Tsuji, 1994), and the far-red fluorescent protein, TurboFP635 (Katushka) (Shcherbo *et al.*, 2010). These have been shown previously to be robust reporters in both pathogenic and non-pathogenic mycobacteria *in vitro* and *in vivo* (Carroll *et al.*, 2010; Parker & Bermudez, 1997; Zelmer *et al.*, 2012). GFP, under control of the constitutive *groEL* promoter, was introduced into a plasmid backbone carrying TurboFP635 under control of a riboswitch-based, theophylline-inducible promoter (Seeliger *et al.*, 2012), to generate the pTiGc FD plasmid (Fig. 1a). Notably, heat-killing of *M. smegmatis *(90 °C, 30 min) resulted in a 15-fold decrease in mean fluorescence intensity when compared with untreated viable bacteria (data not shown), highlighting the suitability of GFP as a marker for cell viability.

### The replication reporter protein is tightly regulated and stable

**Fig. 2.**
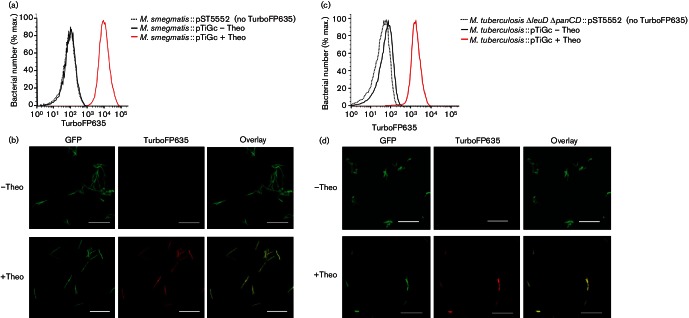
The theophylline-inducible, riboswitch-based promoter allows tight regulation of the FD reporter protein, TurboFP635. *M. smegmatis*::pTiGc was cultured in the presence or absence of 2 mM theophylline (Theo), then analysed by (a) flow cytometry or (b) confocal microscopy. Similarly, *M. tuberculosis *Δ*leuD* Δ*panCD*::pTiGc was cultured in the presence or absence of 2 mM theophylline, then analysed by (c) flow cytometry or (d) confocal microscopy. Representative examples of three independent experiments are shown, demonstrating negligible background and significant upregulation of TurboFP635 expression upon theophylline addition. Bars, 10 µm. Data shown are representative of three independent biological replicates (each with three technical replicates).

To confirm that the pTiGc plasmid allowed for tightly regulated expression of TurboFP635, we measured green and far-red fluorescence intensity of mycobacteria carrying this plasmid in the presence and absence of the inducer, theophylline. No growth defect (in comparison with WT strains) was observed in the presence or absence of theophylline, indicating that expression of the reporter proteins did not incur an *in vitro* fitness cost (data not shown). To facilitate detailed assessment of the fluorescent properties of large numbers of single bacterial cells, we used a combination of flow cytometry and confocal microscopy. As expected, in the absence of induction, the majority of pTiGc-carrying mycobacteria demonstrated green fluorescence, but no red fluorescence above no-reporter control levels (Fig. 2). We confirmed that induction with 2 mM theophylline resulted in high-intensity far-red fluorescence for both *M. smegmatis *::pTiGc (Fig. 2a, b) and *M. tuberculosis *Δ*leuD* Δ*panCD*::pTiGc (Fig. 2c, d). Confocal microscopy showed normal bacterial morphology and no aggregates for both *M. smegmatis *::pTiGc (Fig. 2b) and *M. tuberculosis *Δ*leuD* Δ*panCD*::pTiGc (Fig. 2d). Flow cytometry and confocal microscopy therefore confirmed constitutive GFP expression and tightly regulated, strongly inducible TurboFP635 expression in both *M. smegmatis *and *M. tuberculosis*.

**Fig. 3.**
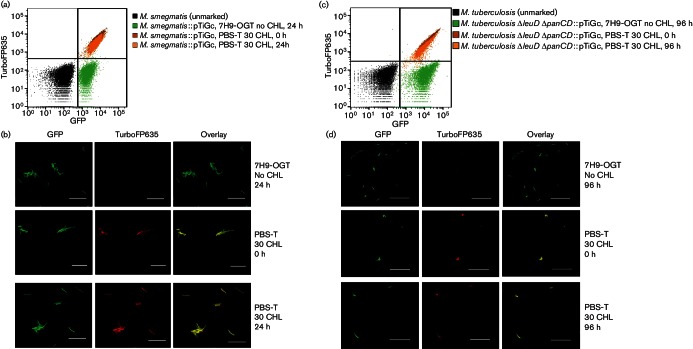
TurboFP635 is sufficiently stable for application as a replication reporter. (a, b) *M. smegmatis*::pTiGc was cultured in 7H9-OGT in the presence of 2 mM theophylline, then passaged twice in PBS-T+2 mM theophylline, before inoculating into PBS-T with no theophylline, containing 30 µg chloramphenicol ml^−1^ (CHL). Samples were analysed by (a) flow cytometry or (b) confocal microscopy. In (a), fluorescence intensity before and after chloramphenicol treatment is depicted by the brown and orange populations, respectively. For comparison, an actively dividing population, 24 h after theophylline withdrawal, is shown in green. The black population represents unmarked bacteria. (c, d) *M. tuberculosis *Δ*leuD* Δ*panCD*::pTiGc was cultured in 7H9-OGT in the presence of 2 mM theophylline, then passaged twice in 7H9-OGT without pantothenate+2 mM theophylline, before inoculating into 7H9-OGT without pantothenate with no theophylline, containing 30 µg chloramphenicol ml^−1^ (CHL). Samples were analysed by (c) flow cytometry or (d) confocal microscopy. In (c), fluorescence intensity before and after chloramphenicol treatment is depicted by the brown and orange populations, respectively. For comparison, an actively dividing population, 96 h after theophylline withdrawal, is shown in green. The black population represents unmarked bacteria. Bars, 10 µm. Data shown are representative of three independent biological replicates (each with three technical replicates).

An important requirement for successful application of the FD technique is stability of the reporter proteins. Specifically, the replication reporter needs to have a sufficiently long half-life such that only bacterial division (and not protein degradation) will result in reduction of signal over the course of the experimental period. To establish if this was indeed the case, we measured reporter protein stability in non-replicating, nutrient-starved cultures in the presence of chloramphenicol, a bacteriostatic drug which blocks *de novo *protein synthesis. Pre-induced *M. smegmatis *::pTiGc cultures were incubated in PBS/0.05 % tyloxapol with 2 mM theophylline, then passaged into PBS/0.05 % tyloxapol with no theophylline in the presence of 30 µg chloramphenicol ml^−1^ for 24 h (Fig. 3a, b). Similarly, pre-induced *M. tuberculosis *::pTiGc cultures were incubated in medium with theophylline, but lacking pantothenate (to restrict growth), before passage into fresh medium with no theophylline or pantothenate, but including 30 µg chloramphenicol ml^−1^ for up to 4 days (Fig. 3c, d). Subsequent analysis by flow cytometry and confocal microscopy confirmed that non-replicating, chloramphenicol-treated mycobacteria retained high levels of green and red fluorescence intensity. We also demonstrated that the TurboFP635 reporter was stable under acidic conditions, as might be encountered within the macrophage environment (Fig. S2). These results indicate that the TurboFP635 reporter is sufficiently stable to be used as a reliable marker of mycobacterial replication.

### FD provides a quantitative measure of mycobacterial replication

**Fig. 4.**
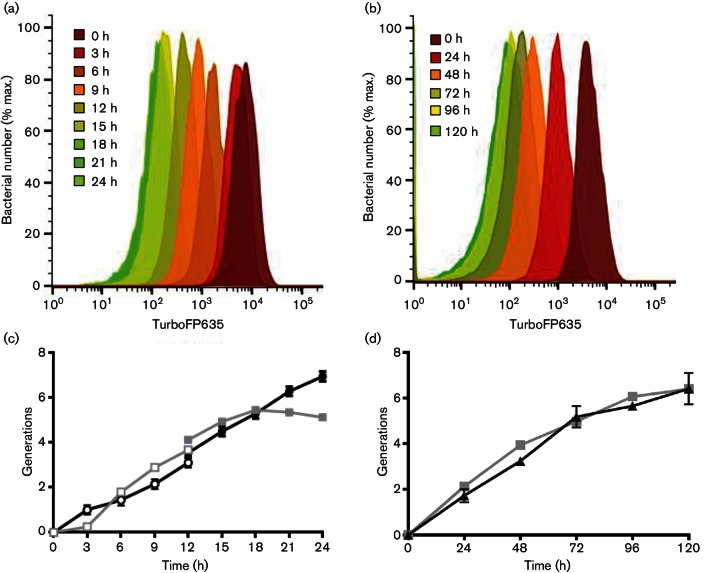
FD provides an accurate measure of mycobacterial generation time. Mycobacterial cultures were cultured in the presence of 2 mM theophylline, then washed and passaged into fresh medium without theophylline, and growth was monitored by OD or c.f.u. and FD. (a, b) Flow cytometric detection of TurboFP635 fluorescence is shown at selected time points for (a) *M. smegmatis *::pTiGc and (b) *M. tuberculosis *Δ*leuD* Δ*panCD*::pTiGc. (c, d) Bacterial generation numbers were calculated from OD (black line) and fluorescence intensity (grey line) measurements for *M. smegmatis *::pTiGc and from CFU (black line) and fluorescence intensity (grey line) measurements for *M. tuberculosis* Δ*leuD* Δ*panCD*::pTiGc as detailed in Methods, and are compared for *M. smegmatis*::pTiGc and *M. tuberculosis *Δ*leuD* Δ*panCD*::pTiGc in (c) and (d), respectively. To enable measurement of *M. smegmatis *growth over 24 h, staggered culture start times were used, with one set of cultures being monitored over the 0–12 h period (open symols) and the second being monitored over the 12–24 h period (filled symbols). Data shown in (c) and (d) are depicted as mean±sd of three technical replicates, and are representative of three independent experiments.

We next wished to determine whether FD could provide a reliable quantitative measure of mycobacterial replication comparable with commonly used methods such as OD measurement. To do this, we passaged pre-induced mycobacteria into medium without theophylline, and incubated with shaking at 37 °C. In this setting, no new TurboFP635 would be synthesized, and therefore in bacteria undergoing active replication, we would expect the mean far-red fluorescence signal to be reduced with each successive cell division. *M. smegmatis *::pTiGc and *M. tuberculosis *Δ*leuD* Δ*panCD*::pTiGc samples were taken at regular intervals for OD and/or c.f.u. measurements and flow cytometry analysis. Flow cytometry data indicated FD during the course of bacterial growth. The change in the geometric mean of far-red fluorescence intensity over time (Fig. 4a, b) was then used to calculate the number of generations and compared with the number of generations calculated from OD or c.f.u. measurements. This demonstrated an excellent correlation up to at least five generations, for both *M. smegmatis *(Fig. 4c) and *M. tuberculosis *(Fig. 4d). While we cannot rule out the possibility that degradation of the TurboFP635 reporter may contribute to the loss of fluorescence, our data suggest otherwise. We note that Pearson correlation tests revealed a statistically significant positive correlation between generations calculated on the basis of OD or c.f.u. and those calculated on the basis of FD for *M. smegmatis *(*r*=0.9394, *N*=9, two-tailed *P* value=0.0002) and *M. tuberculosis *(*r*=0.9908, *N*=6, two-tailed *P* value=0.0001). The mean generation time (based on FD measurements) calculated for *M. smegmatis *during logarithmic growth in rich medium was approximately 3 h 20 min, corresponding to published replication rates (Gill *et al.*, 2009). Similar results were obtained for *M. tuberculosis *Δ*leuD* Δ*panCD*::pTiGc, although with longer calculated generation times (approximately 18 h 43 min), as expected for this slow-growing organism (Gill *et al.*, 2009). These data confirm that FD could be used reliably to monitor mycobacterial replication, for at least five generations.

**Fig. 5.**
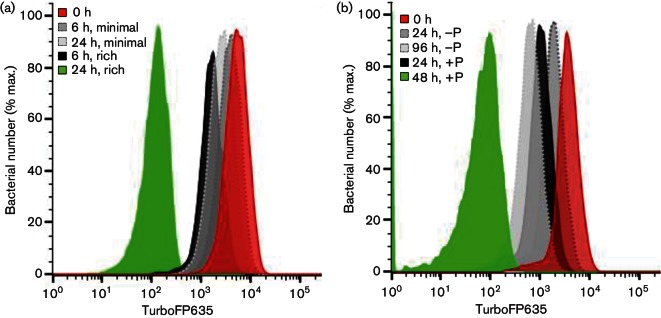
FD can be exploited to assess mycobacterial replication dynamics under different *in vitro *conditions. Mycobacterial cultures were grown in the presence of 2 mM theophylline, then washed and passaged into fresh rich medium without theophylline, and growth was monitored by FD. (a) *M. smegmatis *::pTiGc was passaged into either minimal medium (PBS/0.05 % tyloxapol, dark grey and light grey) or rich medium (7H9-OGT, black and green). Samples were taken at 6 h and 24 h. (b) Similarly, *M. tuberculosis *Δ*leuD* Δ*panCD*::pTiGc was passaged into 7H9-OGT with leucine, and with (black and green, 24 h and 96 h, respectively) or without (dark and light grey, 24 h and 96 h, respectively) pantothenate (P), and flow cytometry samples were taken at 24 and 96 h following withdrawal of theophylline. Data shown are representative of three independent biological replicates (each with three technical replicates).

We went on to validate the use of mycobacterial FD to probe mycobacterial replication dynamics under different *in vitro* conditions. We first examined *M. smegmatis* growth during nutrient limitation. Pre-induced *M. smegmatis *::pTiGc cultures were passaged twice in PBS/0.05 % tyloxapol with 2 mM theophylline to remove extracellular nutrients and deplete intracellular nutrient stores. Nutrient-depleted bacteria were washed to remove theophylline, and then added to PBS/0.05 % tyloxapol (nutrient-limited) or standard nutrient-replete growth medium (7H9-OGT), in the absence of theophylline. For *M. smegmatis, *flow cytometry analysis at 0, 6 and 24 h revealed only a small shift in fluorescence intensity in nutrient-limited medium (PBS/0.05 % tyloxapol) between 0 and 24 h (Fig. 5a). This shift corresponded to <1 generation (0.93±0.3 at 24 h), contrasting with the large shift in the nutrient-rich (7H9-OGT) medium, which corresponded to 5.34±0.15 generations at 24 h.

We performed a similar analysis for *M. tuberculosis*, but here we took advantage of the auxotrophic nature of the *M. tuberculosis *Δ*leuD* Δ*panCD* strain (Sampson *et al.*, 2004). This strain grows normally when supplemented exogenously with leucine and pantothenate, but does not grow in the absence of either of these supplements. *M. tuberculosis *Δ*leuD* Δ*panCD *::pTiGc was first cultured overnight in 7H9-OGT containing 2 mM theophylline (to induce TurboFP635 expression) and supplemented with leucine and pantothenate (to permit growth). Bacteria were washed, then incubated overnight in 7H9-OGT with no pantothenate (to deplete any intracellular stores of this compound), in the presence of 2 mM theophylline. OD measurements confirmed growth restriction (data not shown). The pre-induced, nutrient-starved bacteria were then passaged into 7H9-OGT, supplemented with leucine, without theophylline and with 0 or 24 µg pantothenate ml^−1^. Samples were taken for OD measurements and flow cytometry at the time points indicated (Fig. 5b). As expected, pantothenate depletion restricted growth rates; for pantothenate-depleted cultures after 4 days, FD-calculated generations were 2.78±0.40. In contrast, FD-calculated generations for nutrient-replete cultures were 6.15±0.026 at the same time point (96 h). Together, these results demonstrate that FD can be used reliably to monitor mycobacterial replication, and can provide an accurate measurement of different growth rates.

### Intracellular mycobacterial replication dynamics

**Fig. 6.**
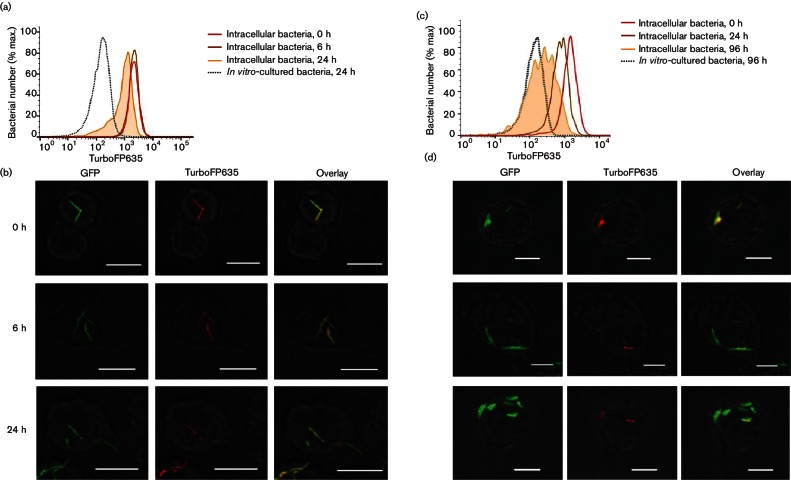
The dual reporter system offers insights into intracellular mycobacterial replication dynamics. RAW264.7 macrophages were infected with *M. smegmatis *::pTiGc (a, b) or *M. tuberculosis *Δ*leuD* Δ*panCD*::pTiGc (c, d), and intracellular mycobacterial replication was compared with that of *in vitro*-cultured mycobacteria, by monitoring TurboFP635 fluorescence. Fluorescence was assessed by flow cytometry (a, c) and confocal microscopy (b, d). Bars, 10 µm. Data shown are representative of three independent biological replicates (each with three technical replicates).

An important potential application for the FD system is to enable the measurement of intracellular mycobacterial replication (or lack thereof). To assess whether this was feasible, we infected murine macrophages with pre-induced *M. smegmatis *::pTiGc. Infected macrophages were lysed, and the mycobacteria recovered were analysed by flow cytometry or c.f.u determination (Fig. 6a). In addition, infected macrophages on coverslips were analysed by confocal microscopy (Fig. 6b). Flow cytometry demonstrated a shift in the geometric mean of far-red fluorescence intensity of GFP-positive *M. smegmatis *recovered from macrophages 24 h post-infection (Fig. 6a), suggesting replication of intracellular *M. smegmatis *::pTiGc. Subsequently, flow cytometric data were used to quantify *M. smegmatis *replication dynamics during macrophage infection. When considering the geometric mean of all far-red fluorescent *M. smegmatis *within the GFP-positive (live) population, the generation number calculated over 24 h was 1.59±0.38, suggestive of minimal replication of intracellular bacteria. This was significantly lower than that of *M. smegmatis *cultured extracellularly in D10 (5.61±1.09). Interestingly, closer analysis of flow cytometric data revealed the presence of a sub-population (22.0±8.8 %) of bacteria with reduced TurboFP635 signal at 24 h (Figs 6a and S3); this was also evident by confocal microscopy (Fig. 6b). We observed a clear shoulder on the left of the asymmetric peak representing intracellular bacteria at 24 h, when compared with intracellular and *in vitro*-cultured bacteria at 0 h (Fig. 6a). When the far-red^HI^ and far-red^LO^ populations were considered independently (Fig. S3), their generation numbers were 0.94±0.12 and 3.39±0.16, respectively. *M. smegmatis *is reported to be rapidly cleared from infected macrophages (Prakash *et al.*, 2010), and c.f.u. results for this experiment are consistent with this (generation number over 24 h <1). However, the flow cytometry data are suggestive of the presence of a sub-population of replicating *M. smegmatis *::pTiGc within the macrophage culture. No extracellular bacteria were observed by confocal microscopy. We acknowledge that streptomycin can affect both extracellular and intracellular bacteria; however, in this study all intracellular bacteria were subjected to the same streptomycin exposure times; therefore, any differences observed between these samples cannot be attributed to the antibiotic. The dual fluorescence reporter system has therefore provided new insights into *M. smegmatis–*macrophage interaction, highlighting the heterogeneous nature of the intracellular mycobacterial population in this context.

### *M. tuberculosis* population heterogeneity and drug-tolerant bacteria (persister) enrichment

We next applied the dual reporter system to interrogate replication dynamics of *M. tuberculosis *during macrophage infection. RAW264.7 macrophages were infected with pre-induced *M. tuberculosis *::Δ*leuD* Δ*panCD *::pTiGc as described above (Fig. 6c, d). Flow cytometry was used to compare the bacterial population recovered from macrophages with those grown *in vitro *for up to 96 h following infection. This revealed that intracellular mycobacteria showed a relatively homogeneous population structure at early time points, with a similar replication rate at 24 h (0.89±0.04) to the *in vitro*-cultured bacteria (0.78±0.15) (Fig. 6c). However, from 48 h onwards, a more heterogeneous population distribution emerged, with a marked slower-growing population at 96 h.

**Fig. 7.**
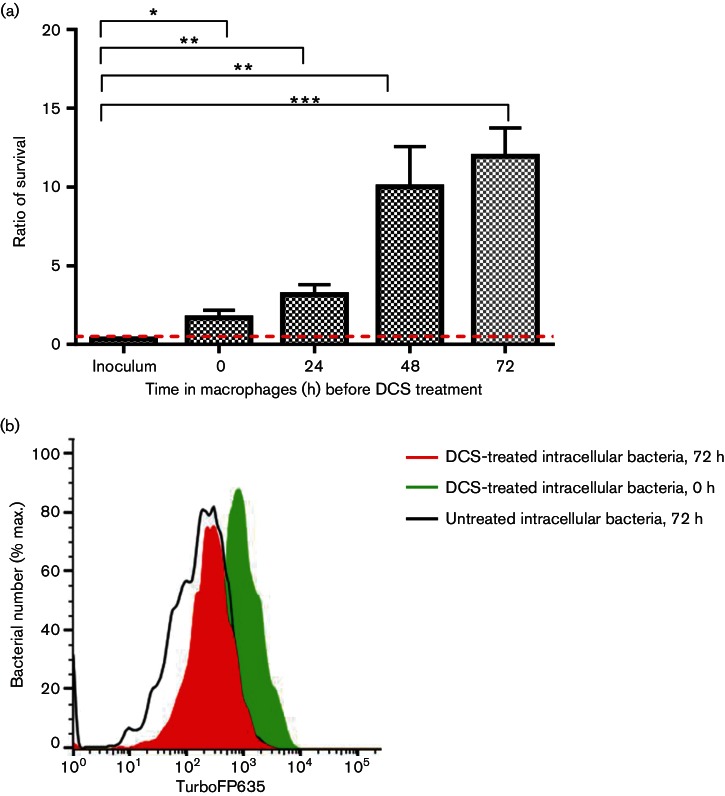
FD allows identification of macrophage-induced persister formation. (a) *M. tuberculosis* Δ*leuD* Δ*panCD*::pTiGc cultured *in vitro* or recovered from RAW264.7 macrophages at different time points post-uptake were transferred to 7H9-OGT, containing 100 µg DCS ml^−1^and incubated for 96 h. Formation of macrophage-induced persisters was measured as the ratio of the percentage of the macrophage-exposed population surviving DCS treatment to the percentage of the *in vitro*-cultured bacteria surviving DCS treatment. Data shown are depicted as mean±sd of three technical replicates, and are representative of three independent experiments. (b) For flow cytometry, RAW264.7 macrophages were infected with *M. tuberculosis* Δ*leuD* Δ*panCD*::pTiGc and treated with 100 µg DCS ml^−1^ for 96 h. Intracellular mycobacterial replication was compared with that of untreated intracellular mycobacteria, by monitoring TurboFP635 fluorescence. Data were analysed by using a Student’s *t-*test. *P<0.05, **P<0.005, ***<0.0005. Data shown are representative of three independent biological replicates (each with three technical replicates).

To determine whether the heterogeneous intracellular *M. tuberculosis *population included persister bacteria, we applied a combination of c.f.u. determination and mycobacterial FD in conjunction with DCS treatment to characterize the mycobacteria recovered from macrophages. A previous study demonstrated that internalization of *Salmonella* by bone-marrow-derived macrophages (BMDM) rapidly induced the formation of persisters (Helaine *et al.*, 2014). Similarly, we observed a significant increase in the proportion of drug-tolerant bacteria (persisters) following internalization by macrophages (Fig. 7a). Interestingly, we observed a significant increase in macrophage-induced drug-tolerant bacteria (persisters) at later time points, suggesting slow adaptive responses to the intracellular microenvironment. Flow cytometry demonstrated that DCS treatment of intracellular bacteria enriched for a high-intensity far-red population with an evidently slower replication rate from 24 h (Fig. 7b). This further suggests that the increasing proportion of slow or non-replicating drug-tolerant bacteria represent the persisters isolated by c.f.u. survival assays over time following macrophage uptake.

## Discussion

A major stumbling block in understanding the biology of latent *M. tuberculosis *infection and the phenomenon of drug tolerance in mycobacteria is the paucity of tools to isolate and characterize persistent mycobacteria. These populations are commonly suggested to exist in a non- or slowly replicating state. However, while there is some evidence to support very slow or no growth of *M. tuberculosis* in human samples (Colangeli *et al.*, 2014; Garton *et al.*, 2008; Walter *et al.*, 2015), results from mice (Gill *et al.*, 2009) and nonhuman primates (Ford *et al.*, 2011) have been interpreted to suggest otherwise. Although several studies have linked *M. tuberculosis* drug tolerance to dormancy/lack of replication (Deb *et al.*, 2009; Rodriguez *et al.*, 2014), other data from *in vitro* studies (Wakamoto *et al.*, 2013) and macrophage models (Adams *et al.*, 2011; Raffetseder *et al.*, 2014) suggest that lack of replication is not an absolute requirement for *M. tuberculosis* drug tolerance. These apparently discrepant results have been derived using diverse experimental approaches, highlighting the need for a new, systematic approach to the problem. Understanding the physiological state of ‘dormant' bacteria, and the cues that induce entry into and exit from a dormant state in clinically latent *M. tuberculosis* infection has important implications for TB treatment and prevention. However, our knowledge of the physiological state of viable, but non-replicating mycobacteria is severely limited, particularly *in vivo*. This is largely due to lack of suitable tools to identify, isolate and characterize non- or slowly replicating mycobacteria. These populations are inherently difficult to isolate, and new tools to do so are urgently required.

We report here on the development and validation of a powerful new tool for monitoring population-wide mycobacterial replication at the single-cell level. FD technology was developed by Helaine *et al.* (2010, 2014) to monitor *Salmonella* replication dynamics, and has yielded striking new insights into intracellular replication dynamics of pathogenic bacteria. Here, we have successfully adapted and applied FD to *M. smegmatis* and *M. tuberculosis*, demon strating its utility in both fast-growing saprophytic and slow-growing pathogenic mycobacteria.

The ability to use FD to accurately quantify bacterial replication dynamics has several prerequisites, including sufficiently bright and stable fluorescent reporter proteins. Here, we have demonstrated that the far-red reporter TurboFP635 is sufficiently bright and stable, and thus we selected this for further experiments. Notably, during optimization of promoter/reporter combinations, we observed that GFP was unexpectedly too unstable to serve as an accurate reporter for mycobacterial replication (data not shown). A second critical feature is that the inducible promoter needs to have undetectable basal activity (Mitchison & Coates, 2004), but must be expressed at high levels upon induction. We found the recently described theophylline-inducible, riboswitch-based promoter (Seeliger *et al.*, 2012) to be highly suited for this application.

In this study, we have demonstrated that the pTiGc dual-colour reporter plasmid can be applied successfully to monitor *in vitro *and intracellular mycobacterial replication dynamics by FD. We have shown that we can accurately measure at least five bacterial generations. This is comparable with previous work using FD in *Salmonella* (Helaine *et al.*, 2010), as well as with studies using microfluidics platforms to monitor single-cell replication dynamics of *M. smegmatis *(Aldridge *et al.*, 2012). Future versions of this system could include a second inducible promoter, an approach that has been applied successfully in *Salmonella* to extend the number of measurable generations up to 10 (Helaine *et al.*, 2010).

The pTiGc reporter system could easily be combined with FACS to isolate different populations for downstream analysis. Until now, this has not been feasible, and previous studies have relied on methods such as preferential lysis of replicating bacteria using antibiotics or other chemical treatment (Canas-Duarte *et al.*, 2014; Keren *et al.*, 2011). Alternatively, analyses have been performed on enriched, but still potentially heterogeneous, populations (Betts *et al.*, 2002; Deb *et al.*, 2009; Gengenbacher *et al.*, 2010; Mak *et al.*, 2012). The dual reporter system described here offers the ability to rapidly and specifically identify non- or slowly replicating mycobacteria in physiologically relevant environments. In combination with FACS, this system will allow the purification of non-replicating bacteria from other populations, enabling subsequent characterization of relatively homogeneous populations.

Application of the dual reporter system to characterize intracellular *M. smegmatis *revealed that while the majority of bacteria were non or slowly replicating, a subpopulation of apparently dividing *M. smegmatis *could be detected. Numerous reports indicate that *M. smegmatis *does not survive within murine macrophages (Prakash *et al.*, 2010), with the underlying assumption that it is unable to grow in this environment. However, these studies rely on c.f.u. enumeration as the primary assay for mycobacterial survival. This provides only a summative measure of the growth rate, and does not take into account population heterogeneity. As highlighted by Anes *et al.* (2006) even non-pathogenic *M. smegmatis* can undergo periods of intracellular replication. Our findings support this, and highlight the ability of the dual reporter system to provide a window into unexpected mycobacterial population heterogeneity.

Our dual reporter system also revealed population heterogeneity for intracellular *M. tuberculosis*. Interestingly, early time points showed relatively homogeneous replication rates, very similar to those of *in vitro-*cultured bacteria. However, as the infection proceeded, a more slowly replicating population emerged, suggesting a programmed adaptation to the intracellular environment. This is consistent with a recent study which reported a decrease in replication rate and ribosomal activity of *M. tuberculosis* in the later stages of a murine infection model (Manina *et al.*, 2015). Transcriptional analyses have demonstrated that *M. tuberculosis *undergoes metabolic remodelling upon exposure to the host environment, and during drug treatment of infected hosts (Rodriguez *et al.*, 2014; Walter *et al.*, 2015); the FD system described here provides a powerful tool to explore the heterogeneity of this remodelling and its impact on intracellular growth of mycobacteria. Determination of the physiological state and long-term fate of the slowly replicating population would be of great interest.

We have shown that FD could be applied to demonstrate an increase in the proportion of non- or slowly replicating drug-tolerant bacteria (likely persisters) upon exposure to the intracellular macrophage environment, as revealed by DCS treatment. This corresponds with a previous study which showed that internalization of *Salmonella* by BMDM resulted in the formation of persisters. However, in that study a significant increase in macrophage-induced persisters was observed after only 15 min, representing a much more rapid response than seen in the present study. Interestingly, our results suggest that longer internalization of mycobacteria by macrophages resulted in a significant increase in non-replicating persisters at later time points (72 and 96 h), compared with earlier time points (24 and 48 h). A recent study has indicated that mycobacterial phenotypic variation is the result of host immunity and antibiotic stress (Manina *et al.*, 2015), while others have reported that physical confinement induces drug tolerance (Luthuli *et al.*, 2015). However, the precise mechanisms by which these populations are induced are currently still unknown. Acidification and nutrient deprivation within the *Salmonella-*containing vacuole have been shown to play a role in the phenotypic heterogeneity and persister formation observed (Helaine *et al.*, 2014). It remains to be determined whether similar signals encountered within the phagolysosomal compartment trigger mycobacterial persister formation. Additionally, the effect of immunological activation of macrophages on *M. tuberculosis *persister formation warrants further investigation. However, we note that the activation of BMDMs with IFN-γ when infected with *Salmonella* did not result in any change in persister proportions (Helaine *et al.,* 2014).

Variants of the dual reporter system described here could be combined with other types of mycobacterial biosensors, such as those for monitoring intracellular redox potential (Bhaskar *et al.*, 2014), pH and chloride levels (Tan *et al.*, 2013), to enable the simultaneous measurement of defined physiological characteristics. In combination with technologies such as flow cytometry, microfluidics and high-resolution microscopy, this will allow us to probe the impact of bacterial microenvironment on replication dynamics and phenotypic heterogeneity.

In summary, we have demonstrated that FD can be successfully applied both *in vitro *and in macrophage infection models, offering unique opportunities to study how environmental stressors and the innate immune response might impact on phenotypes such as drug tolerance. Ultimately, this system could find application in whole-animal infection models, to better understand the host and bacterial signals which lead to mycobacterial persistence.
